# Early-Phase Trajectory of Right Ventricular Hemodynamics during Microaxial Flow Pump Support for Cardiogenic Shock

**DOI:** 10.1016/j.jacadv.2026.103165

**Published:** 2026-08-19

**Authors:** Yuki Ikeda, Keita Saku, Jun Nakata, Takashi Unoki, Shohei Nakahara, Toshiyuki Iwaya, Saeko Iikura, Yu Takigami, Takeshi Yamamoto, Tomohiro Sakamoto, Junya Ako, UNLOADERS-PVAD Investigators

**Affiliations:** a Department of Cardiovascular Medicine, Kitasato University School of Medicine, Sagamihara, Kanagawa, Japan; b Department of Cardiovascular Dynamics, National Cerebral and Cardiovascular Center, Suita, Osaka, Japan; c Division of Cardiovascular Intensive Care, Nippon Medical School Hospital, Bunkyo-Ku, Tokyo, Japan; d Division of Cardiology, Saiseikai Kumamoto Hospital Cardiovascular Center, Kumamoto, Japan

**Keywords:** percutaneous ventricular assist device, pulmonary artery catheterization, pulmonary artery pulsatility index, right atrial pressure, right ventricular dysfunction

## Abstract

**Background:**

Right ventricular dysfunction (RVD) is common in cardiogenic shock and associated with poor outcomes. However, early RVD trajectories after initiation of percutaneous ventricular assist device (PVAD) support and their prognostic implications remain unclear.

**Objectives:**

The objectives of the study was to characterize the trajectory of hemodynamic RVD (hRVD) during the first 24 hours after PVAD initiation and evaluate its association with outcomes.

**Methods:**

This study included patients with cardiogenic shock treated with PVAD from the UNLOADERS-PVAD registry. RVD was hemodynamically defined by an elevated right atrial pressure ≥15 mm Hg and/or a low pulmonary artery pulsatility index <0.9. Hemodynamic assessments were performed pre-PVAD, early post-PVAD, and 24 h post-PVAD. The primary endpoint was a composite of all-cause mortality or reintroduction of mechanical circulatory support after PVAD discontinuation within 30 days.

**Results:**

Among 463 patients with available data, hRVD was present in 52% (63/121) pre-PVAD, decreased to 48% (211/443) early post-PVAD, and further to 39% (169/436) at 24 h post-PVAD. hRVD trajectories were heterogeneous, with improvement predominating over deterioration. The median follow-up from PVAD implantation was 27 (12–30) days. Persistent or worsening hRVD at 24 h post-PVAD was associated with a higher risk of the primary endpoint. Elevated right atrial pressure was associated with adverse outcomes only at 24 h post-PVAD, whereas low pulmonary artery pulsatility index was consistently associated with adverse outcomes across all time points.

**Conclusions:**

In patients with cardiogenic shock supported by PVAD, the early trajectory of hRVD provides prognostic information. Persistent or worsening hRVD at 24 hours identifies high-risk patients and may guide timely escalation of right-heart-targeted therapies. (UNLOADERS-PVAD [Unloading and Heart Recovery with Advanced Mechanical Circulatory Support: Optimal Management of Percutaneous Ventricular Assist Device] UMIN000052966).

Cardiogenic shock is a life-threatening syndrome with 30-day mortality of 30% to 40%.^1^, ^2^, ^3^, ^4^ For patients with cardiogenic shock refractory to pharmacological therapy, the initiation of mechanical circulatory support (MCS) may be required. The Impella (2.5, CP, 5.0, and 5.5), a left-sided percutaneous ventricular assist device (PVAD), is a microaxial flow pump that provides left ventricular unloading while maintaining end-organ perfusion.^5^ Because PVADs are fundamentally limited to left-sided circulatory support, there is concern that adequate hemodynamic improvement may not be achieved in patients with concomitant right ventricular dysfunction (RVD).^5^ Therefore, understanding the clinical relevance of concomitant RVD in patients receiving PVAD support is an important clinical priority.

RVD is common in advanced heart failure and cardiogenic shock and is strongly associated with adverse outcomes.^6^, ^7^, ^8^, ^9^ Approximately 30% of patients with acute myocardial infarction complicated by cardiogenic shock treated with PVAD exhibit concomitant RVD, which is associated with increased in-hospital mortality.^10^ However, the temporal trajectory of right ventricular function following PVAD initiation and the optimal timing for prognostic risk stratification remain poorly defined. A better understanding of RVD trajectories may enable early identification of high-risk patients who require additional therapies, including escalation to right-heart MCS. Furthermore, trajectory-based assessment may refine patient selection for PVAD by identifying individuals who were not represented in prior randomized controlled trials but may nevertheless benefit from PVAD support.^11^

Hemodynamic indices derived from pulmonary artery catheterization, such as right atrial pressure (RAP) and the pulmonary artery pulsatility index (PAPi), have been proposed as reliable markers for identifying RVD in patients with cardiogenic shock.^12^^,^^13^ The first 24 hours after treatment initiation represent a critical therapeutic window during which treatment response should be assessed to guide further escalation.^14^

We aimed to characterize the early trajectory of RVD during the first 24 hours after PVAD initiation in patients with cardiogenic shock and to examine its prognostic implications.

## Methods

### Study design and participants

This study was conducted as a subanalysis of the UNLOADERS-PVAD (UNLoading and heart recOvery with ADvanced mEchanical ciRculatory Support: optimal management of Percutaneous Ventricular Assist Device) registry.^15^ The UNLOADERS-PVAD registry is a multicenter registry that enrolled consecutive PVAD-treated cardiogenic shock patients at 3 Japanese shock hubs (April 2018–December 2023). Cardiogenic shock was defined as persistent hypotension (systolic blood pressure <90 mm Hg or mean arterial pressure 30 mm Hg lower than at baseline) with severe reduction in cardiac index (<1.8 L/min/m^2^ without support or <2.2 L/min/m^2^ with support) and adequate or elevated filling pressure (left ventricular end-diastolic pressure >15 mm Hg).^16^ We excluded patients in whom RVD could not be assessed at any time point due to missing RAP or PAPi data.

This study was registered with the University hospital Medical Information Network Clinical Trial Registry (UMIN000052966). This study complied with the Declaration of Helsinki and the Japanese Ethical Guidelines for Medical and Health Research Involving Human Subjects, and the study protocol was approved by the ethics committee of the Clinical Research Review Board of the Kitasato Institute (B23-029). An opt-out method was applied, and the requirement for written informed consent was waived because of complete data anonymization and the observational nature of the current registry.

### Data collection and evaluation of right ventricular dysfunction

According to the UNLOADERS-PVAD registry protocol, comprehensive clinical data including vital signs, device flow, hemodynamics, and laboratory data were collected at the following time points: immediately before PVAD initiation (pre-PVAD), the first postinitiation measurement set obtained as soon as feasible after PVAD initiation (early post-PVAD), and 24 hours after PVAD initiation (24 h post-PVAD).^15^ The early post-PVAD data were defined as the earliest time point at which comprehensive clinical data could be obtained within 12 hours after PVAD implantation, and the 24 h post-PVAD data were defined as the measurements closest to 24 hours after PVAD implantation within a window of 12 to 36 hours, as determined by each data abstractor. In patients in whom venoarterial extracorporeal membrane oxygenation (VA-ECMO) was initiated before PVAD implantation, pre-PVAD data were collected under VA-ECMO support. If VA-ECMO was used at any point during PVAD support, the patient was classified as having concomitant VA-ECMO. Baseline data including demographics, medical history, shock etiology, Society for Cardiovascular Angiography and Interventions-Cardiogenic Shock Working Group (SCAI-CSWG) stage, comatose status, mechanical ventilation, concomitant VA-ECMO, cardiac arrest, and intraaortic balloon pump support before PVAD initiation were collected before PVAD initiation. Data on the type of first-line PVAD and PVAD upgrades were obtained from medical records following PVAD implantation. The SCAI-CSWG stages were defined based on a previous study.^17^

There is no universally accepted definition of RVD. Diagnostic criteria are commonly based on echocardiographic evidence of impaired right ventricular systolic function and/or right heart chamber dilation.^18^ In the setting of cardiogenic shock, RVD has also been proposed to be identified using hemodynamic indices.^10^^,^^13^^,^^19^ In this study, RVD was hemodynamically defined using RAP and PAPi and was termed hemodynamic RVD (hRVD), defined as RAP ≥15 mm Hg and/or PAPi <0.9. PAPi was calculated as follows: (systolic pulmonary artery pressure – diastolic pulmonary artery pressure)/RAP.

### Outcome evaluation

The primary endpoint was the first occurrence of all-cause mortality after PVAD implantation or MCS reintroduction. MCS reintroduction was defined as unplanned initiation of VA-ECMO, PVAD, or intraaortic balloon pump after PVAD discontinuation. PVAD discontinuation was defined as removal of the index PVAD. All outcome data were retrospectively obtained from medical records. The primary endpoint was assessed at 30 days after PVAD implantation or at hospital discharge, whichever occurred first. This composite endpoint was selected because both all-cause mortality and MCS reintroduction represent clinically relevant adverse outcomes during the short-term follow-up. We also evaluated the incidence of complications during PVAD support, including hemolysis, major bleeding, and limb ischemia. The definitions of each complication were based on the UNLOADERS-PVAD registry protocol.^15^

### Statistical analysis

All statistical analyses and graphics were performed using R (version 4.4.0; R Foundation for Statistical Computing). A 2-sided *P* value <0.05 was considered statistically significant. Missing data were not imputed, and the number of missing values was reported. Continuous variables are presented as median and IQR. Categorical variables are expressed as numbers and percentages. Group differences were evaluated using the Kruskal-Wallis test for continuous variables, and the Fisher exact test for categorical variables. Transitions in the proportion of patients with hRVD were illustrated using a Sankey diagram. Baseline factors associated with hRVD, RAP ≥15 mm Hg, and PAPi <0.9 at each assessment time point were evaluated using separate univariable logistic regression analyses, and the results are presented as ORs with 95% CIs.

Natural cubic splines with 3 degrees of freedom were used to assess the association between RAP/PAPi and the event rate (%) for the primary endpoint. Internal knots were automatically selected according to the observed data distribution. Time-dependent receiver operating characteristic curves at 30 days were constructed for RAP and PAPi at each assessment time point to evaluate their ability to discriminate the primary endpoint. Time-dependent areas under the curve were estimated, and optimal cutoff values were determined using the Youden index. Cumulative incidence curves for the primary endpoint were estimated using the Kaplan-Meier method and compared using the log-rank test. Time zero was defined as either pre-PVAD or 24 h post-PVAD, as appropriate. The proportional hazards assumption for the Cox proportional hazards models was evaluated using Schoenfeld residuals. Cox proportional hazards models were used to evaluate the association between each variable and the primary endpoint, with the HR and 95% CIs. We did not fit fully adjusted multivariable models for the association between hRVD and outcomes to avoid potential overadjustment and collider bias. Some variables related to shock severity and early management may lie on the causal pathway. Accordingly, we present unadjusted estimates and assessed robustness using prespecified subgroup analyses and interaction testing across clinically relevant strata. For continuous hemodynamic variables, univariable Cox proportional hazards models were used to estimate HRs per 1-SD increase, with corresponding 95% CIs and Wald chi-square statistics. Heterogeneity of HRs was assessed across subgroups defined by age, sex, shock cause, SCAI-CSWG stage, concomitant VA-ECMO, and cardiac arrest, and *P* values for interaction were calculated. As a sensitivity analysis, we additionally fitted an adjusted Cox proportional hazards model to evaluate the association between hRVD at 24 h post-PVAD and the primary endpoint. In addition, cumulative incidence curves were constructed after excluding patients with prior VA-ECMO use before PVAD implantation.

## Results

### Patient characteristics

Of the 501 registry patients, 463 with available RAP and/or PAPi data allowing hRVD assessment at least at 1 time point were included in the analysis (Supplemental Figure 1). The median duration of follow-up from PVAD implantation was 27 (12–30) days in the overall cohort. Baseline characteristics of the study population are summarized in Table 1. The median age was 70 (59–77) years, and most patients were males (335/463, 72%). Myocardial infarction was the most common etiology of cardiogenic shock (278/463, 60%), and more than half of the patients were classified as SCAI-CSWG stage ≥D (260/463, 56%). Concomitant VA-ECMO support was used in 59% (273/463) of patients. The most frequently used PVAD was the Impella CP (381/463, 82%). Among the 273 patients receiving concomitant VA-ECMO, VA-ECMO was initiated before PVAD implantation in 206 patients. Among patients receiving concomitant VA-ECMO, the time difference in initiation relative to PVAD support was −1.0 (−2.2 to −0.5) hours in the VA-ECMO first group, whereas it was +2.2 (+0.8 to +14.0) hours in the PVAD first group.

**Table 1:** Baseline Characteristics (N = 463)

	Missing Data, n (%)	Value
Demographics		
Age, y	0 (0)	70 [59–77]
Male	0 (0)	335 (72)
Height, cm	1 (0)	164 [157–170]
Weight, kg	0 (0)	63 [54–72]
Medical history		
HTN	0 (0)	269 (58)
DM	0 (0)	178 (38)
DLP	0 (0)	173 (37)
Smoker	0 (0)	233 (50)
Shock etiology	0 (0)	
AMI		278 (60)
HF deterioration		86 (19)
Myocarditis		41 (9)
Other		58 (12)
SCAI-CSWG stage ≥D	0 (0)	260 (56)
Systolic BP <90 mm Hg	19 (4)	208 (47)
Mean BP <60 mm Hg	20 (4)	114 (26)
Lactate ≥4 mmol/L	13 (3)	256 (57)
GCS <9	0 (0)	247 (53)
Mechanical ventilation	0 (0)	420 (91)
Concomitant VA-ECMO	0 (0)	273 (59)
Cardiac arrest	0 (0)	
OHCA		102 (22)
IHCA		133 (29)
Type of first-line PVAD	0 (0)	
Impella 2.5		54 (12)
Impella CP		381 (82)
Impella 5.0		10 (2)
Impella 5.5		18 (4)
IABP support prior to PVAD initiation	0 (0)	52 (11)

Values are median (IQR) or n (%).

Vital signs and blood sample data were collected immediately before PVAD initiation.

AMI = acute myocardial infarction; BP = blood pressure; CSWG = cardiogenic shock working group; DLP = dyslipidemia; DM = diabetes mellitus; GCS = Glasgow coma scale; HF = heart failure; HTN = hypertension; IABP = intra-aortic balloon pump; IHCA = in-hospital cardiac arrest; OHCA = out-of-hospital cardiac arrest; PVAD = percutaneous ventricular assist device; SCAI = society for cardiovascular angiography and interventions; VA-ECMO = venoarterial extracorporeal membrane oxygenation.

### Prevalence of hRVD

**Figure 1:**
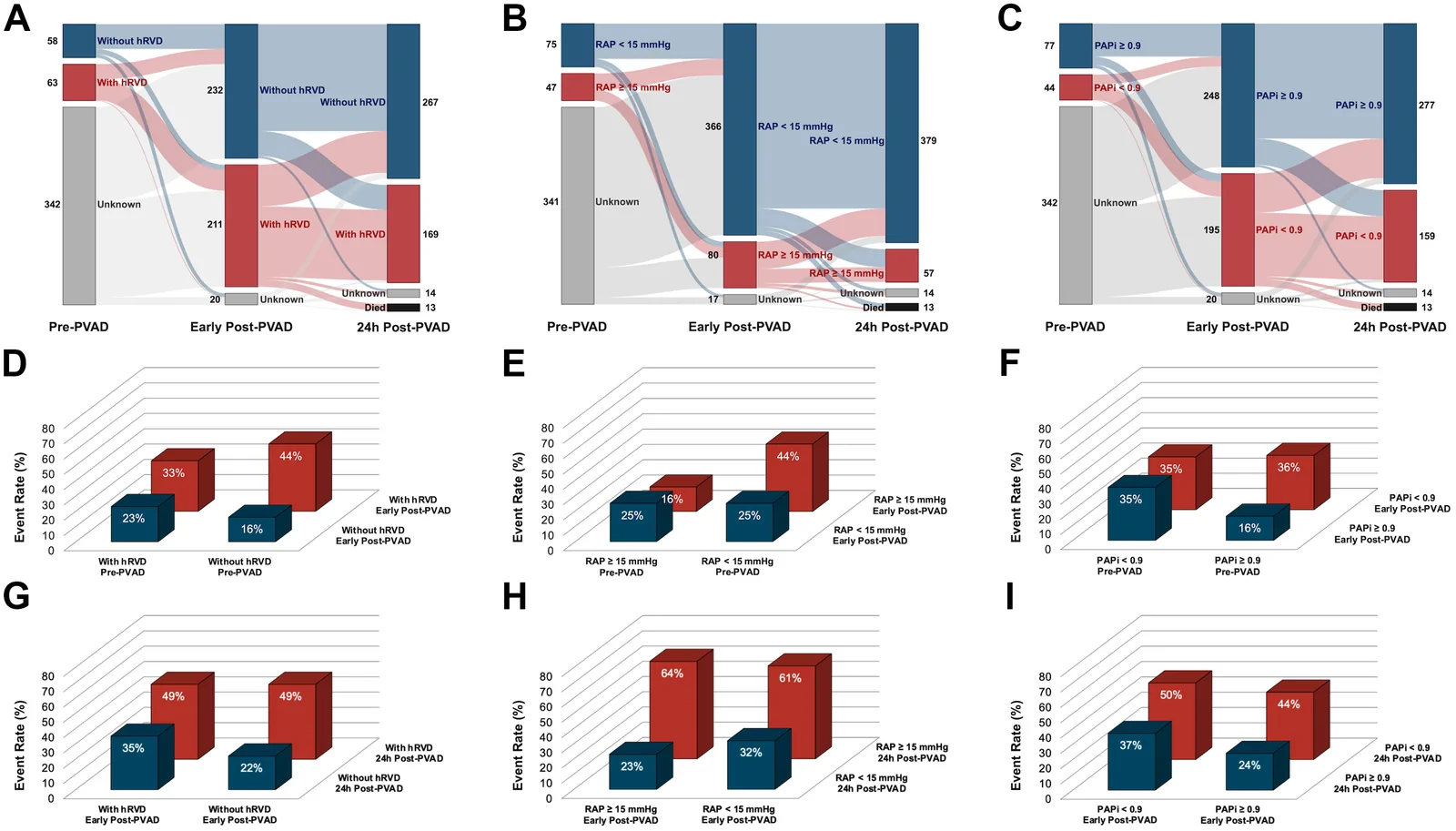
Trajectory of hRVD Across Assessment Time Points and Associated Event Rates Sankey diagrams illustrating transitions in hRVD status (A), RAP (B), and PAPi (C) across assessment time points. Bar charts showing the associations between transitions in hRVD status (D and G), RAP (E and H), and PAPi (F and I) and event rates for the primary endpoint. Bar charts were generated using data from patients with available and evaluable paired measurements at each time point. hRVD = hemodynamic right ventricular dysfunction; PAPi = pulmonary artery pulsatility index; PVAD = percutaneous ventricular assist device; RAP = right atrial pressure.

**Figure 2:**
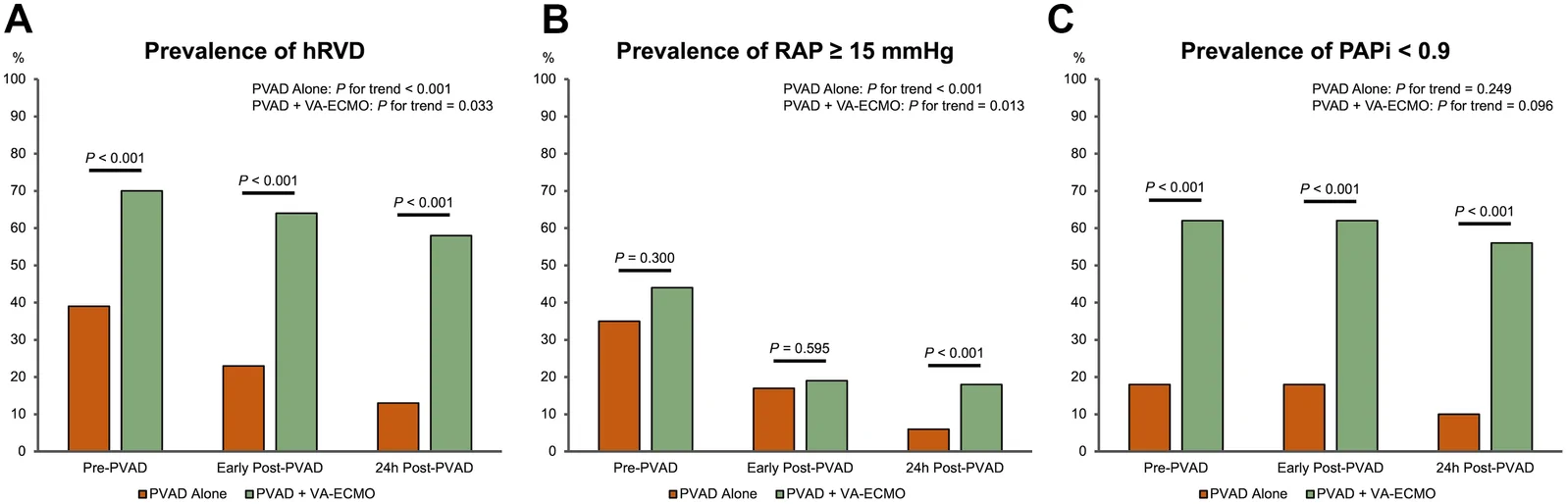
Prevalence of hRVD, Elevated RAP, and Low PAPi Across Assessment Time Points, Stratified by Concomitant VA-ECMO Prevalence of hRVD (A), elevated RAP (B), and low PAPi (C). Prevalence is presented at pre-PVAD, early post-PVAD, and 24 h post-PVAD, stratified by PVAD alone vs concomitant VA-ECMO. Between-group differences at each time point were compared using *P* values. *P* values for trend were calculated among patients with measurements available at all 3 time points. VA-ECMO = venoarterial extracorporeal membrane oxygenation; other abbreviations as in Figure 1.

The prevalence of hRVD, elevated RAP, and low PAPi at each assessment time point is illustrated in Figures 1A to 1C using Sankey diagrams. Among evaluable patients, hRVD was present in 52% (63/121) pre-PVAD, 48% (211/443) early post-PVAD, and 39% (169/436) at 24 h post-PVAD; RAP ≥15 mm Hg in 39% (47/122), 18% (80/446), and 13% (57/436), respectively; and PAPi <0.9 in 36% (44/121), 44% (195/443), and 36% (159/436), respectively. Figure 2 shows the prevalence of hRVD, RAP ≥15 mm Hg, and PAPi <0.9 at each assessment time point, stratified by the presence or absence of concomitant VA-ECMO. The relationship between RAP and PAPi differed according to concomitant VA-ECMO, as shown in Supplemental Figure 2.

### Factors associated with hRVD

Hemodynamic parameters stratified by the presence or absence of hRVD at each assessment time point are presented in Table 2. Missing data were more frequent for cardiac output-derived parameters pre-PVAD and early post-PVAD, whereas few hemodynamic variables were missing at 24 h post-PVAD. At 24 h post-PVAD, the median flow rates in the overall cohort were 2.4 (1.6–3.1) L/min for PVAD support and 3.0 (2.4–3.5) L/min for VA-ECMO support. Compared with patients without hRVD, those with hRVD exhibited lower PVAD flow and higher VA-ECMO flow at 24 h post-PVAD (PVAD flow: 2.7 [2.1-3.3] vs 1.7 [0.8-2.5] L/min, *P* < 0.001; VA-ECMO flow: 2.7 [1.8-3.2] vs 3.1 [2.7-3.6] L/min, *P* < 0.001). Laboratory parameters divided by hRVD status at each assessment time point are summarized in Table 3. As shown in Table 4, age <65 years, female sex, myocarditis as the shock etiology, SCAI-CSWG stage ≥D, concomitant VA-ECMO, and cardiac arrest were each associated with the presence of hRVD at all or some assessment time points.

**Table 2:** Hemodynamic Parameters

	Missing Data, n (%)	All^a^	Groups by RV Metrics Across Phases		*P* Value
			Without hRVD (RAP <15 mm Hg and PAPi ≥0.9)	With hRVD (RAP ≥15 mm Hg and/or PAPi <0.9)	
Pre-PVAD					
Patients, n		121	58	63	
Mean BP, mm Hg	2 (2)	71 (62–80)	71 (60–79)	71 (62–80)	0.763
SAPP, mm Hg	2 (2)	34 (24–41)	34 (26–44)	34 (20–41)	0.208
HR, beats/min	2 (2)	92 (78–115)	93 (78–115)	92 (76–119)	0.900
RAP, mm Hg	0 (0)	12 (9–17)	10 (7–12)	17 (14–21)	<0.001
Mean PAP, mm Hg	0 (0)	28 (22–34)	28 (22–31)	29 (23–37)	0.101
PAPP, mm Hg	0 (0)	17 (10–21)	17 (13–23)	11 (7–20)	0.001
PAPi	0 (0)	1.2 (0.7–1.7)	1.7 (1.3–2.4)	0.7 (0.4–1.0)	<0.001
PAWP, mm Hg	0 (0)	24 (19–28)	21 (16–25)	25 (20–31)	<0.001
RAP/PAWP ratio	0 (0)	0.5 (0.4–0.7)	0.4 (0.3–0.6)	0.6 (0.5–0.8)	<0.001
PVR, WU	29 (24)	1.6 (0.5–2.3)	1.8 (1.1–2.4)	0.9 (0–2.2)	0.020
PA compliance, mL/mm Hg	29 (24)	1.9 (1.3–2.8)	1.8 (1.4–2.5)	2.0 (1.2–3.4)	0.309
PA elastance, mm Hg/mL	29 (24)	1.2 (0.9–1.7)	1.2 (0.9–1.5)	1.3 (0.9–1.9)	0.181
CO, L/min	29 (24)	3.0 (2.3–3.7)	3.0 (2.4–3.9)	3.0 (2.2–3.5)	0.364
LVCPO, W	29 (24)	0.44 (0.33–0.58)	0.46 (0.36–0.69)	0.43 (0.33–0.56)	0.313
RVCPO, W	29 (24)	0.19 (0.13–0.25)	0.17 (0.13–0.26)	0.20 (0.15–0.24)	0.539
RVSWi, g･m/m^2^/beat	29 (24)	3.4 (2.0–6.1)	4.5 (2.8–7.6)	2.8 (1.4–4.2)	<0.001
Early post-PVAD^b^					
Patients, n		443	232	211	
RAP, mm Hg	0 (0)	10 (6–13)	7 (5–10)	13 (9–16)	<0.001
Mean PAP, mm Hg	0 (0)	19 (13–25)	19 (14–25)	18 (13–24)	0.419
PAPP, mm Hg	0 (0)	10 (5–16)	14 (10–19)	6 (4–10)	<0.001
PAPi	0 (0)	1.0 (0.6–2.0)	2.0 (1.3–2.9)	0.5 (0.3–0.7)	<0.001
PAWP, mm Hg	0 (0)	15 (11–20)	14 (10–18)	15 (11–21)	0.001
RAP/PAWP ratio	0 (0)	0.6 (0.5–0.8)	0.5 (0.3–0.6)	0.8 (0.6–1.0)	<0.001
PVR, WU	74 (17)	2.0 (1.0–3.1)	2.2 (1.2–3.1)	1.8 (0.8–3.2)	0.049
CO, L/min	74 (17)	2.3 (1.1–3.6)	3.1 (2.0–4.2)	1.4 (0.5–2.7)	<0.001
RVCPO, W	74 (17)	0.11 (0.04–0.19)	0.15 (0.08–0.21)	0.06 (0.02–0.13)	<0.001
24 h post-PVAD					
Patients, n		436	267	169	
Mean BP, mm Hg	0 (0)	76 (69–85)	77 (70–86)	75 (66–82)	0.008
SAPP, mm Hg	0 (0)	22 (6–38)	30 (15–44)	8 (3–24)	<0.001
HR, beats/min	0 (0)	78 (66–90)	80 (70–92)	73 (60–85)	<0.001
RAP, mm Hg	0 (0)	9 (6–12)	7 (5–10)	12 (10–15)	<0.001
Mean PAP, mm Hg	0 (0)	19 (15–24)	20 (15–24)	18 (14–22)	0.004
PAPP, mm Hg	0 (0)	12 (7–16)	14 (11–18)	6 (4–8)	<0.001
PAPi	0 (0)	1.2 (0.6–2.2)	2.0 (1.3–3.1)	0.5 (0.2–0.7)	<0.001
PAWP, mm Hg	0 (0)	14 (11–18)	13 (10–17)	15 (12–20)	<0.001
RAP/PAWP ratio	0 (0)	0.6 (0.4–0.8)	0.5 (0.4–0.6)	0.7 (0.6–0.9)	<0.001
PVR, WU	6 (1)	1.7 (0.9–2.7)	1.7 (1.1–2.6)	1.5 (0–3.2)	0.005
PA compliance, mL/mm Hg	15 (1)	2.7 (1.7–3.8)	2.7 (1.9–3.7)	2.7 (1.4–5.0)	0.920
PA elastance, mm Hg/mL	10 (1)	0.8 (0.6–1.6)	0.7 (0.5–1.0)	1.4 (0.8–2.4)	<0.001
CO, L/min	6 (1)	2.7 (0.5–4.1)	3.5 (2.4–4.5)	1.0 (0.5–2.7)	<0.001
LVCPO, W	6 (1)	0.47 (0.12–0.71)	0.62 (0.42–0.78)	0.17 (0.07–0.42)	<0.001
RVCPO, W	6 (1)	0.12 (0.03–0.19)	0.16 (0.09–0.22)	0.04 (0.02–0.11)	<0.001
RVSWi, g·m/m^2^/beat	12 (1)	2.7 (0.5–5.0)	4.2 (2.5–6.3)	0.5 (0.1–1.5)	<0.001

Values are median (IQR) or n (%).

At 24 h post-PVAD, 13 patients who died and 1 patient who underwent early device removal were not evaluated.

PVR = (mean PAP – PAWP)/CO; PA compliance = (CO/HR)/(systolic PAP – diastolic PAP); PA elastance = systolic PAP/(CO/HR); LVCPO = (CO × mean BP)/451; RVCPO = (CO × mean PAP)/451; RVSWi = (CO/[HR × BSA]) × (mean PAP − RAP) × 0.0136.

CO = cardiac output; HR = heart rate; hRVD = hemodynamic right ventricular dysfunction; LVCPO = left ventricular cardiac power output; PA = pulmonary artery; PAP = pulmonary artery pressure; PAPi = pulmonary artery pulsatility index; PAPP = pulmonary artery pulse pressure; PAWP = pulmonary artery wedge pressure; PVR = pulmonary vascular resistance; RAP = right atrial pressure; RV, right ventricular; RVCPO = right ventricular cardiac power output; RVSWi = right ventricular stroke work index; SAPP = systemic artery pulse pressure; other abbreviations as in Table 1.

a Patients in whom the presence or absence of hRVD could be evaluated.

b Mean BP, SAPP, and HR were not available at the early post-PVAD.

**Table 3:** Blood Sample Parameters

	Missing Data, n (%)	All^a^	Groups by RV Metrics Across Phases		*P* Value
			Without hRVD (RAP <15 mm Hg and PAPi ≥0.9)	With hRVD (RAP ≥15 mm Hg and/or PAPi <0.9)	
Pre-PVAD					
Patients, n		121	58	63	
pH	1 (1)	7.3 (7.3–7.4)	7.3 (7.3–7.4)	7.4 (7.3–7.4)	0.610
HCO3-, mmol/L	1 (1)	20 (17–23)	20 (17–23)	20 (16–23)	0.186
Lactate, mmol/L	1 (1)	2.3 (1.5–4.5)	2.2 (1.4–4.0)	2.4 (1.7–5.2)	0.322
T-bil, mg/dL	0 (0)	0.9 (0.5–1.5)	0.7 (0.5–1.2)	1.0 (0.7–1.8)	0.012
ALT, U/L	0 (0)	45 (21–123)	33 (19–59)	86 (24–195)	0.001
BUN, mg/dL	0 (0)	25 (19–39)	22 (18–35)	28 (20–44)	0.037
Cr, mg/dL	0 (0)	1.3 (0.9–2.1)	1.1 (0.8–1.6)	1.5 (1.0–2.4)	0.020
24 h post-PVAD					
Patients, n		436	267	169	
pH	0 (0)	7.4 (7.3–7.4)	7.4 (7.3–7.4)	7.3 (7.3–7.4)	<0.001
HCO3-, mmol/L	0 (0)	22 (20–24)	22 (20–24)	21 (19–23)	0.088
Lactate, mmol/L	0 (0)	1.8 (1.2–2.9)	1.5 (1.2–2.5)	2.3 (1.4–3.4)	<0.001
T-bil, mg/dL	1 (0)	1.6 (1.0–2.3)	1.5 (1.0–2.3)	1.7 (1.0–2.4)	0.360
ALT, U/L	1 (0)	87 (40–192)	71 (29–151)	121 (61–234)	<0.001
BUN, mg/dL	0 (0)	28 (20–40)	28 (21–41)	26 (19–38)	0.060
Cr, mg/dL	0 (0)	1.3 (0.9–2.2)	1.3 (0.9–2.3)	1.2 (0.8–2.1)	0.041

Values are median (IQR) or n (%).

At 24 h post-PVAD, 13 patients who died and 1 patient who underwent early device removal were not evaluated.

ALT = alanine aminotransferase; BUN = blood urea nitrogen; Cr = creatinine; HCO3- = bicarbonate; T-bil = total bilirubin; other abbreviations as in Tables 1 and 2.

a Patients in whom the presence or absence of hRVD could be evaluated.

**Table 4:** Baseline Factors Associated With hRVD

	hRVD Pre-PVAD		hRVD Early Post-PVAD		hRVD 24 h Post-PVAD	
	Unadjusted OR	*P* Value	Unadjusted OR	*P* Value	Unadjusted OR	*P* Value
Age <65 y	2.18 (1.05-4.51)	0.035	1.65 (1.11-2.46)	0.011	1.99 (1.33-2.98)	<0.001
Female	1.57 (1.03-2.39)	0.035	1.57 (1.03-2.39)	0.035	1.32 (0.86-2.03)	0.195
Shock etiology: myocarditis	4.10 (1.90-8.87)	<0.001	4.10 (1.90-8.87)	<0.001	2.08 (1.06-4.08)	0.031
SCAI-CSWG stage ≥D	1.59 (1.09-2.33)	0.016	1.59 (1.09-2.33)	0.016	2.66 (1.77-4.00)	<0.001
Concomitant VA-ECMO	5.87 (3.82-9.02)	<0.001	5.87 (3.82-9.02)	<0.001	9.49 (5.83-16.02)	<0.001
Cardiac arrest: yes	1.96 (1.34-2.87)	<0.001	1.96 (1.34-2.87)	<0.001	2.32 (1.56-3.45)	<0.001

	**RAP ≥15 mm Hg Pre-PVAD**		**RAP ≥15 mm Hg Early Post-PVAD**		**RAP ≥15 mm Hg 24 h Post-PVAD**	
	**Unadjusted OR**	***P* Value**	**Unadjusted OR**	***P* Value**	**Unadjusted OR**	***P* Value**
Age <65 y	2.09 (1.00-4.44)	0.049	1.46 (0.89-2.39)	0.135	1.90 (1.08-3.33)	0.027
Female	0.51 (0.22-1.13)	0.097	1.72 (1.02-2.86)	0.042	1.53 (0.83-2.73)	0.165
Shock etiology: myocarditis	1.49 (0.62-3.60)	0.370	3.15 (1.55-6.24)	0.002	1.57 (0.28-1.64)	0.328
SCAI-CSWG stage ≥D	1.72 (0.83-3.62)	0.147	1.08 (0.66-1.78)	0.753	2.20 (1.21-4.16)	0.008
Concomitant VA-ECMO	1.47 (0.70-3.11)	0.301	1.14 (0.70-1.90)	0.594	3.47 (1.81-7.25)	<0.001
Cardiac arrest: yes	0.69 (0.29-1.57)	0.380	0.87 (0.53-1.41)	0.577	1.22 (0.70-2.15)	0.477

	**PAPi <0.9 Pre-PVAD**		**PAPi <0.9 Early Post-PVAD**		**PAPi <0.9 24 h Post-PVAD**	
	**Unadjusted OR**	***P* Value**	**Unadjusted OR**	***P* Value**	**Unadjusted OR**	***P* Value**
Age <65 y	2.36 (1.11-5.11)	0.025	1.85 (1.25-2.75)	0.002	2.06 (1.37-3.10)	<0.001
Female	0.58 (0.26-1.30)	0.189	1.53 (1.00-2.33)	0.048	1.38 (0.90-2.13)	0.142
Shock etiology: myocarditis	3.83 (1.57-9.74)	0.003	4.16 (2.04-9.19)	<0.001	2.33 (1.19-4.61)	0.013
SCAI-CSWG stage ≥D	1.37 (0.65-2.89)	0.409	1.75 (1.19-2.58)	0.004	2.96 (1.95-4.53)	<0.001
Concomitant VA-ECMO	7.28 (3.25-17.2)	<0.001	7.57 (4.84-12.2)	<0.001	11.5 (6.84-20.5)	<0.001
Cardiac arrest: yes	1.87 (0.83-4.23)	0.129	2.25 (1.54-3.32)	<0.001	2.38 (1.60-3.57)	<0.001

Values are OR (95% CI) for association with hRVD.

Abbreviations as in Tables 1 and 2.

### Outcomes

**Figure 3:**
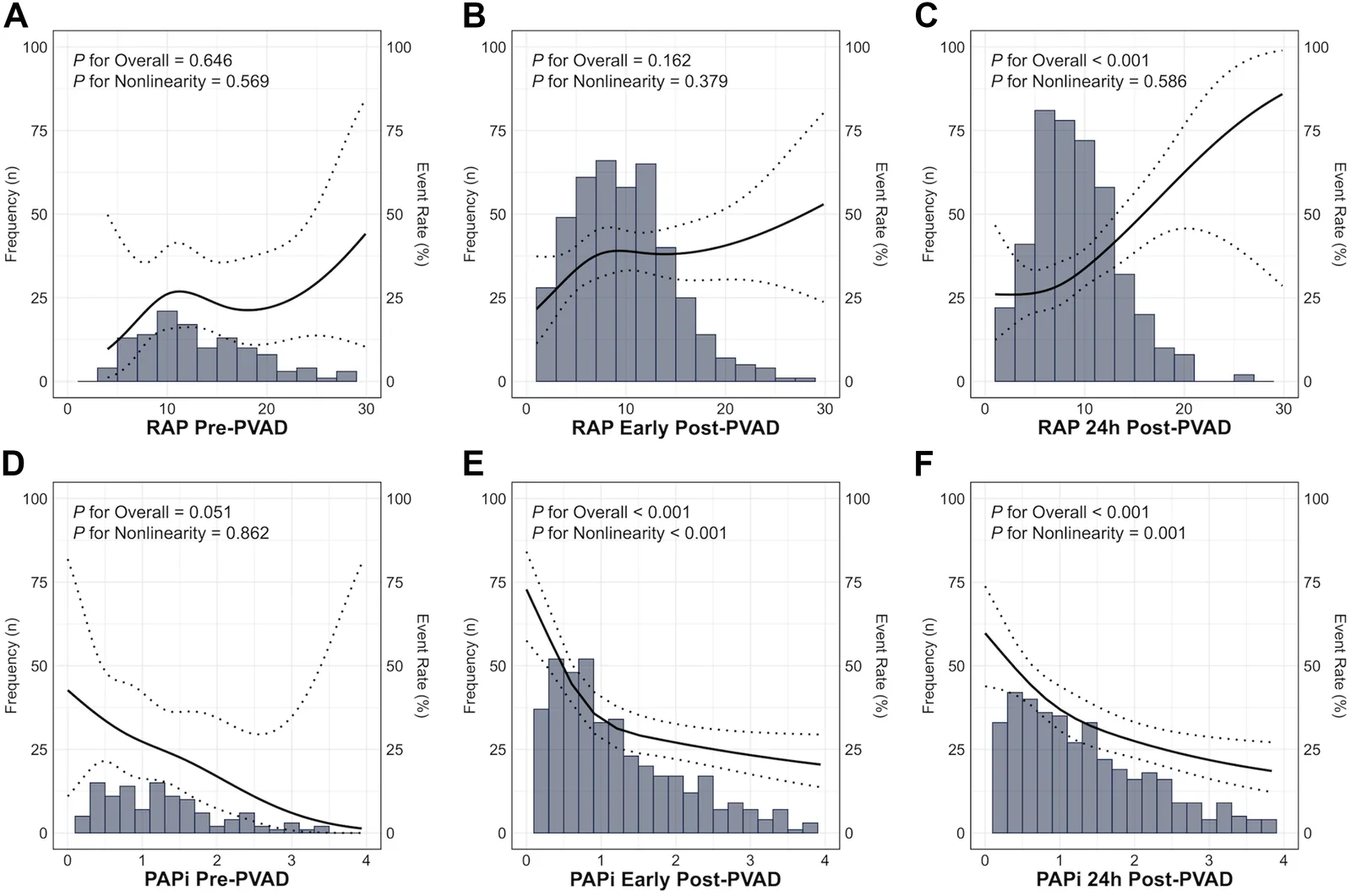
Associations of RAP and PAPi With Event Rates Across Assessment Time Points Spline curves showing the relationships between RAP (A to C) and PAPi (D to F) and the event rates of the primary endpoint at each assessment time point. Abbreviations as in Figure 1.

**Figure 4:**
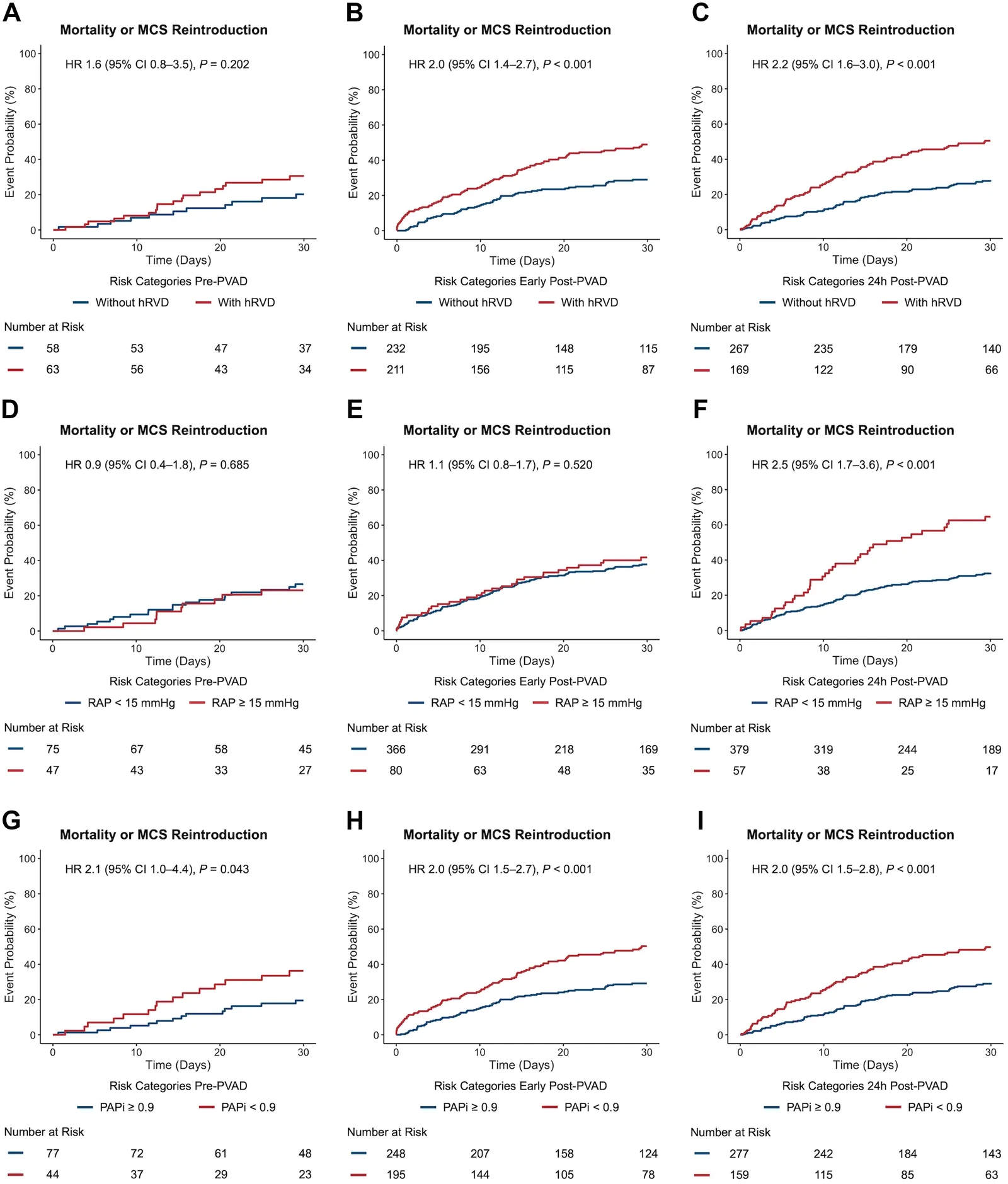
Cumulative Incidence of the Primary Endpoint According to hRVD, RAP, and PAPi Kaplan-Meier curves for the cumulative incidence of the primary endpoint stratified by hRVD status (A to C), RAP (D to F), and PAPi (G to I) at each assessment time point. MCS reintroduction was defined as unplanned initiation of VA-ECMO, PVAD, or intraaortic balloon pump after PVAD discontinuation. MCS = mechanical circulatory support; other abbreviations as in Figure 1.

**Figure 5:**
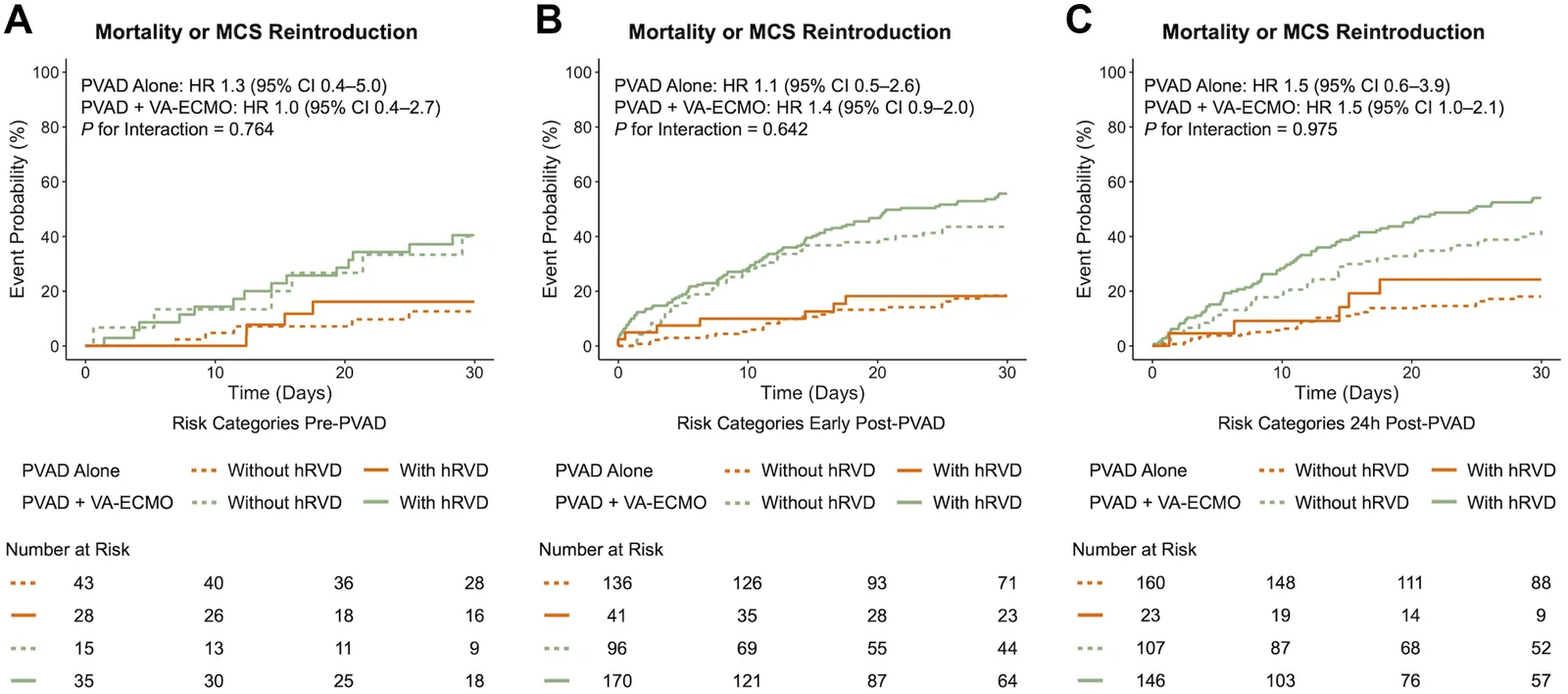
Cumulative Incidence of the Primary Endpoint According to hRVD, Stratified by Concomitant VA-ECMO Kaplan-Meier curves for the cumulative incidence of the primary endpoint stratified by hRVD status, with comparisons between PVAD alone and concomitant VA-ECMO use at each assessment time point (A to C). *P* values for interaction between hRVD status and concomitant VA-ECMO use are presented. MCS reintroduction was defined as unplanned initiation of VA-ECMO, PVAD, or intraaortic balloon pump after PVAD discontinuation. Abbreviations as in Figures 1, 2, and 4.

The associations between transitions in hRVD status, RAP, and PAPi and event rates for the primary endpoint are shown in Figures 1D to 1I. The proportional hazards assumption was not violated for any of the Cox proportional hazards models evaluating the association between hRVD and the primary endpoint (global *P* = 0.94 for pre-PVAD, 0.68 for early post-PVAD, and 0.50 for 24-hour post-PVAD). Figure 3 (overall cohort) and Supplemental Figure 3 (stratified by concomitant VA-ECMO) illustrate the relationships between RAP and PAPi at each assessment time point and the event rates of the primary endpoint using spline curves. Supplemental Figure 4 shows time-dependent receiver operating characteristic curves of RAP and PAPi at each assessment time point for discrimination of the primary endpoint. Figure 4 presents Kaplan-Meier curves for the cumulative incidence of the primary endpoint stratified by hRVD status, RAP, and PAPi. The presence of hRVD was associated with a higher incidence of the primary endpoint, with the strongest association observed at 24 h post-PVAD. Elevated RAP was not significantly associated with the primary endpoint pre-PVAD or early post-PVAD, but was significantly associated with the primary endpoint at 24 h post-PVAD. In contrast, a lower PAPi was consistently and significantly associated with the primary endpoint across all assessment time points. Figure 5 shows Kaplan-Meier curves evaluating the association between hRVD and the primary endpoint, stratified by PVAD alone vs concomitant VA-ECMO. There was no significant heterogeneity in this association according to concomitant VA-ECMO. In a sensitivity analysis adjusted for age, sex, acute myocardial infarction as the shock etiology, and SCAI-CSWG stage ≥D, hRVD at 24 h post-PVAD remained associated with a higher risk of the primary endpoint (adjusted HR: 2.0; 95% CI: 1.4-2.8; *P* < 0.001). Even when the analysis was restricted to patients without prior VA-ECMO use before PVAD implantation, the association between hRVD and the cumulative incidence of the primary endpoint remained consistent with the main analysis, as shown in Supplemental Figure 5. At 24 h post-PVAD, the presence of hRVD was associated with longer durations of VA-ECMO and PVAD support compared with the absence of hRVD (VA-ECMO: 7.0 [4.8-9.9] vs 5.3 [2.7-6.9] days, *P* < 0.001; PVAD: 8.9 [5.8-14.4] vs 6.1 [3.8-9.6] days, *P* < 0.001).

Supplemental Figure 6 presents subgroup analyses evaluating whether the HR associated with hRVD for the primary endpoint differed according to baseline characteristics. Little heterogeneity was observed, except for an interaction by sex for hRVD at 24 h post-PVAD. The strength of the associations between right-heart hemodynamic parameters, including their changes, and the primary endpoint at each time point is summarized in Table 5. At 24 h post-PVAD, RAP demonstrated strong associations with the primary endpoint, whereas PAPi showed a consistently strong association across all assessment time points. Among the changes in hemodynamic parameters, only the change in RAP from pre-PVAD to early post-PVAD was associated with the primary endpoint. The associations of RAP-PAPi categories with the primary endpoint across assessment time points are shown in Supplemental Table 1. Supplemental Table 2 summarizes the associations of hRVD, RAP ≥15 mm Hg, and PAPi <0.9 with mortality when mortality alone was considered as the outcome. RAP ≥15 mm Hg at 24 h post-PVAD was associated with a higher risk of mortality, whereas PAPi <0.9 remained consistently associated with a higher risk of mortality across all assessment time points. The relationships between hRVD status and complications during PVAD support are summarized in Supplemental Table 3. Although hemolysis tended to be more frequent in patients with hRVD, the difference was not statistically significant. Major bleeding was more common in patients with hRVD.

**Table 5:** Associations Between Right Heart Hemodynamic Parameters and the Primary Endpoint

	Pre-PVAD			Early Post-PVAD			24 h Post-PVAD		
Variable	Unadjusted HR	Wald Chi-Square Value	*P* Value	Unadjusted HR	Wald Chi-Square Value	*P* Value	Unadjusted HR	Wald Chi-Square Value	*P* Value
RAP	1.18 (0.83-1.68)	0.86	0.352	1.14 (0.98-1.32)	3.00	0.083	1.33 (1.16-1.53)	16.25	<0.001
PAPi	0.47 (0.25-0.87)	5.73	0.017	0.58 (0.41-0.82)	9.38	0.002	0.69 (0.52-0.90)	7.36	0.007
RAP/PAWP ratio	1.53 (1.12-2.09)	7.22	0.007	1.01 (0.87-1.17)	0.02	0.892	1.25 (1.08-1.43)	9.65	0.002
PVR	1.53 (1.07-2.18)	5.35	0.021	1.10 (0.96-1.26)	1.88	0.170	1.12 (0.99-1.27)	3.37	0.066

	Change in Variables From Pre-PVAD to Early Post-PVAD			Change in Variables From Early Post-PVAD to 24 h Post-PVAD		
Variable	Unadjusted HR	Wald Chi-Square Value	*P* Value	Unadjusted HR	Wald Chi-Square Value	*P* Value
%ΔRAP	1.42 (1.04-1.95)	4.79	0.029	1.10 (0.96-1.26)	1.97	0.160
%ΔPAPi	0.75 (0.45-1.26)	1.17	0.279	1.03 (0.90-1.18)	0.22	0.638
%ΔRAP/PAWP ratio	1.00 (0.67-1.50)	0.00	0.993	1.04 (0.90-1.20)	0.29	0.592
%ΔPVR	0.57 (0.25-1.27)	1.91	0.167	1.03 (0.87-1.22)	0.12	0.726

Values are HRs per 1-SD increase with 95% CIs. Wald chi-square statistics and corresponding *P* values were obtained from univariable Cox proportional hazards models.

PVR (WU) = (mean PAP – PAWP)/CO.

Changes in variables were analyzed in patients with available and evaluable data at each time point.

Abbreviations as in Tables 1 and 2.

## Discussion

In this multicenter, registry-based subanalysis, we investigated the early trajectory of hRVD in patients with cardiogenic shock supported by PVAD (Central Illustration). The main findings were as follows. First, approximately half of the patients had hRVD at PVAD initiation, but hRVD status changed substantially over the first 24 hours, showing bidirectional transitions, with improvement observed in a greater proportion of patients. Second, hRVD was consistently associated with adverse clinical outcomes, with the strongest prognostic impact observed at 24 hours after PVAD initiation. Third, elevated RAP showed an association only at 24 hours after PVAD initiation, whereas lower PAPi showed a robust and consistent association with poor outcomes across all assessment time points.

**Central Illustration:**
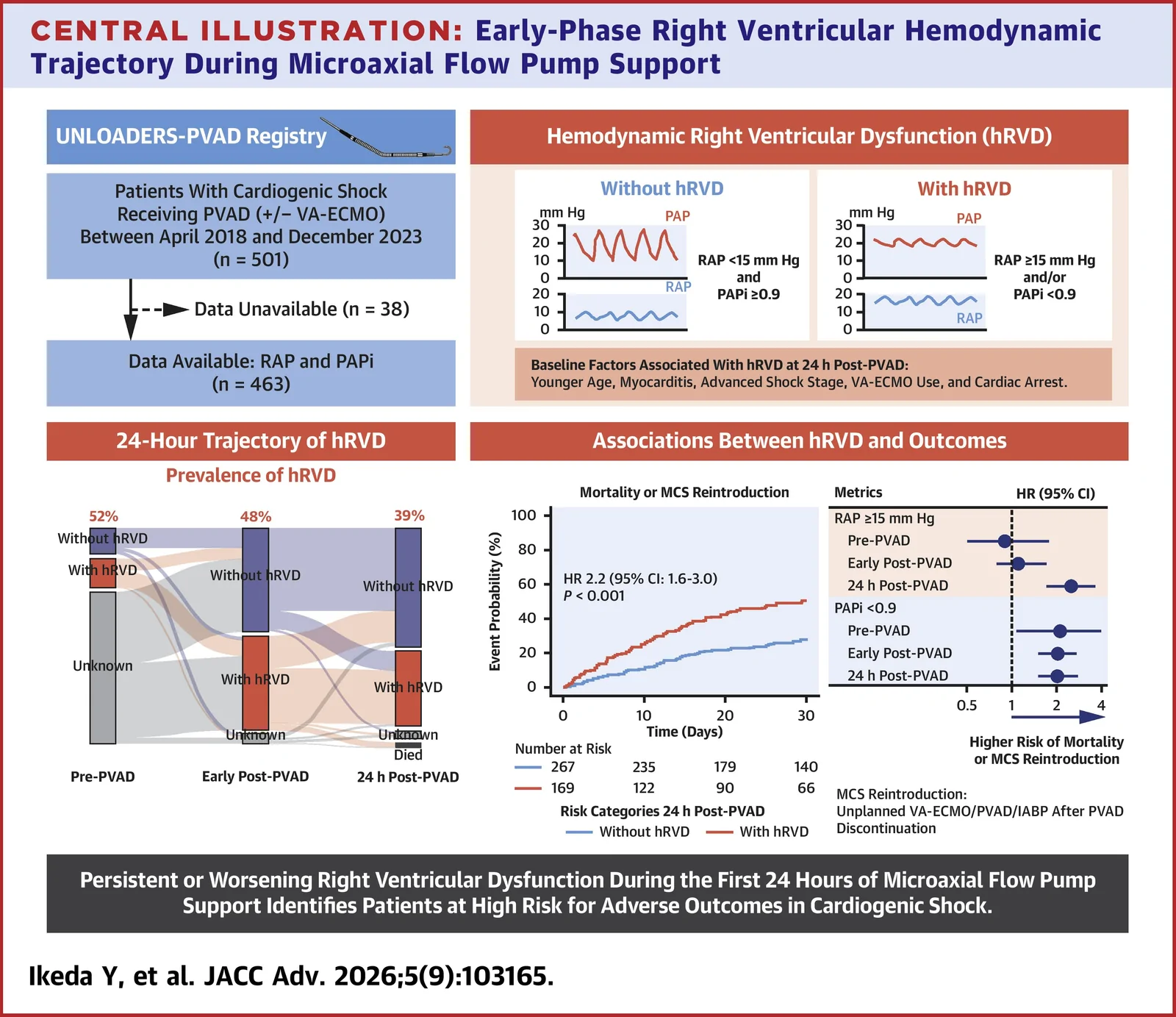
Early-Phase Right Ventricular Hemodynamic Trajectory During Microaxial Flow Pump Support IABP = intraaortic balloon pump; MCS = mechanical circulatory support; PAP = pulmonary artery pressure; PAPi = pulmonary artery pulsatility index; PVAD = percutaneous ventricular assist device; RAP = right atrial pressure; VA-ECMO = venoarterial extracorporeal membrane oxygenation.

This is the first study to systematically evaluate the temporal trajectory of RVD before and after PVAD initiation and to link these dynamic changes with short-term clinical outcomes. By incorporating serial assessments during the first 24 hours before and after PVAD initiation, our results provide novel insights into the optimal timing for patient risk stratification to guide additional intervention for RVD during PVAD support.

hRVD trajectories over 24 hours were heterogeneous; failure to improve by 24 hours was associated with worse outcomes. This study is consistent with prior reports linking RVD to poor outcomes in cardiogenic shock and further explores the temporal trajectory of RVD.^8^^,^^10^ The impact of RVD on clinical outcomes in patients with PVAD support is likely multifactorial. Potential mechanisms include impaired systemic circulatory support due to suction events of PVAD or inadequate left ventricular filling, acute kidney injury related to hemolysis, and progressive end-organ congestion.^10^^,^^20^, ^21^, ^22^, ^23^ In this study, major bleeding was identified as a complication associated with hRVD, whereas hemolysis showed a trend toward higher incidence but did not reach statistical significance.

The early improvement in hRVD observed after PVAD initiation likely reflects favorable changes in ventricular interdependence and pulmonary circulation mediated by effective left ventricular unloading.^5^^,^^12^^,^^24^ In addition, these findings may have been influenced by concomitant fluid management and potential spontaneous improvement in right ventricular function. Our findings further indicate that reassessment of right ventricular hemodynamics at 24 hours after PVAD initiation represents a critical inflection point for risk stratification. Although early-phase hRVD that is reversible may reflect transient right ventricular afterload mismatch or acute volume redistribution, persistent or worsening of hRVD at 24 hours more likely indicates intrinsic RVD or sustained right ventricular-pulmonary arterial uncoupling that is unlikely to recover with left ventricular unloading alone.^12^^,^^19^ This highlights the importance of trajectory-based assessment. In this study, the trajectory of hRVD categories, rather than changes in individual parameters, appeared more relevant for risk stratification. No cutoff values superior to existing thresholds were identified, and current hRVD definitions may remain useful for clinical decision-making despite the influence of concomitant VA-ECMO.

RAP and PAPi showed distinct prognostic implications. RAP reflects right ventricular filling pressure and venous congestion and is highly susceptible to acute changes in volume status and MCS settings.^12^^,^^25^^,^^26^ In contrast, PAPi integrates both pulmonary artery pulsatility and RAP, thereby capturing right ventricular-pulmonary arterial coupling.^12^^,^^25^, ^26^, ^27^ The consistent association between low PAPi and adverse outcomes across all time points suggests that PAPi may serve as a more robust marker of intrinsic RVD in the setting of cardiogenic shock.

The PAPi was proposed as a clinical indicator reflecting right ventricular systolic function and has been reported to be useful for identifying right ventricular failure after inferior acute myocardial infarction as well as for predicting right ventricular failure following left ventricular assist device implantation.^19^^,^^28^^,^^29^ In addition, low PAPi has been shown to serve as an effective prognostic stratification marker in patients with advanced heart failure and those with cardiogenic shock.^30^^,^^31^ The present findings are consistent with these prior reports and further suggest that PAPi represents a robust and universal outcome stratification marker even during the dynamic early-phase of PVAD therapy for cardiogenic shock.

Our findings suggest that reassessment of right ventricular hemodynamics at 24 hours after PVAD initiation represents a critical decision point. Patients with persistent or worsening hRVD at this time may benefit from intensified right-heart intervention, including optimization of preload and afterload of right ventricle or consideration of escalation to right-heart MCS.

Acute right ventricular failure in cardiogenic shock requires a comprehensive approach targeting right ventricular preload, afterload, contractility, and heart rate.^26^ Preload reduction may be achieved with diuretic agents or renal replacement therapy, afterload reduction with vasodilators, and contractility augmented with inotropic agents, and heart rate control with pacemakers applied when indicated.^26^ When these interventions are insufficient, escalation to right-heart MCS should be considered.^27^ In patients supported with PVAD alone, escalation to VA-ECMO or other right-heart MCS may help prevent progressive end-organ congestion while facilitating right ventricular unloading.^27^ In patients receiving concomitant VA-ECMO, additive therapies such as inhaled nitric oxide or transition from VA-ECMO to devices specifically designed for right ventricular support have been reported to promote VA-ECMO weaning.^27^^,^^32^ The present findings support reassessment at 24-hour intervals to guide such decisions and suggest that RAP and PAPi are useful hemodynamic markers for identifying high-risk patients. However, the optimal adjunctive therapies for these patients remain to be determined.

### Study limitations

Limitations include a retrospective design and a modest sample size. Missing data were present for several hemodynamic variables. Therefore, potential bias related to nonrandom missingness cannot be excluded. RVD in this study was defined using hemodynamic indices derived from pulmonary artery catheterization, specifically RAP and PAPi. Because many prior studies have primarily defined RVD using echocardiographic parameters, the interpretation of our results should take into account differences in the definition and assessment of RVD.^18^ Moreover, echocardiographic measures of right ventricular function were not available in this study, precluding direct evaluation of correlations between hemodynamic and echocardiographic indices. The influence of VA-ECMO flow on the interpretation of RAP and PAPi also warrants consideration. Because VA-ECMO provides active drainage from the right atrium, RAP and PAPi may be substantially influenced, and caution is therefore required when interpreting these parameters in patients receiving concomitant VA-ECMO. In this study, the relationship between RAP and PAPi appeared to differ according to the presence or absence of concomitant VA-ECMO, suggesting that the absolute values of PAPi may not be directly comparable between patients supported with PVAD alone and those receiving concomitant VA-ECMO. Nevertheless, subgroup analyses demonstrated no significant heterogeneity in the association between hRVD and clinical outcomes according to concomitant VA-ECMO. Finally, subgroup analyses across key baseline characteristics showed no substantial heterogeneity in the association between hRVD and outcomes, although residual confounding by unmeasured or incompletely measured variables remains possible.

## Conclusions

In patients with cardiogenic shock supported by PVAD, the early trajectory of hRVD provides important prognostic information. Persistent or worsening hRVD at 24 hours after PVAD initiation, particularly as assessed by RAP and PAPi, identifies patients at high risk for adverse outcomes and may help guide timely escalation of right-heart-targeted therapies.

Perspectives **COMPETENCY IN MEDICAL KNOWLEDGE AND PATIENT CARE:** This study highlights the importance of monitoring RVD using RAP and PAPi in patients with cardiogenic shock supported by PVAD. Monitoring the early 24-hour trajectory of right ventricular hemodynamics following PVAD initiation enables timely risk stratification and guides additional interventions.

**TRANSLATIONAL OUTLOOK:** Right-heart-targeted interventions should incorporate a comprehensive approach that includes both pharmacologic strategies and optimization or escalation of MCS. Future studies should clarify effective interventions for cardiogenic shock complicated by RVD, with the goal of improving outcomes following PVAD support.

## Funding support and author disclosures

Dr Ikeda has received honoraria from J&J MedTech and Mallinckrodt Pharma K.K. Dr Saku has received research funding from J&J MedTech, NTT Research, Inc, Asahi Kasei ZOLL Medical Corporation, Neuroceuticals Inc., and Zeon Medical Inc; and honoraria from J&J MedTech and Mallinckrodt Pharma K.K. Dr Nakata has received honoraria from J&J MedTech. Dr Ako has received honoraria from J&J MedTech. All other authors have reported that they have no relationships relevant to the contents of this paper to disclose.
