# Supplementary material for: Early-Phase Trajectory of Right Ventricular Hemodynamics during Microaxial Flow Pump Support for Cardiogenic Shock
Source: JACC Adv. 2026 Aug 19;5(9):103165. doi: 10.1016/j.jacadv.2026.103165 (PMC13521089; doi:10.1016/j.jacadv.2026.103165)
Supplement: Supplementary file 1 [file mmc1.pdf]

Supplemental Appendix

Early-Phase Trajectory of Right Ventricular Hemodynamics During Microaxial Flow Pump Support for Cardiogenic Shock

Contents

Supplemental Tables…………………………………………………………………………………2

Supplemental Table 1……………………………………………………………………2

Supplemental Table 2……………………………………………………………………3

Supplemental Table 3……………………………………………………………………4

Supplemental Figures..………………………………………………………………………………5

Supplemental Figure 1..…………………………………………………………………5

Supplemental Figure 2..…………………………………………………………………6

Supplemental Figure 3..…………………………………………………………………7

Supplemental Figure 4..…………………………………………………………………8

Supplemental Figure 5..…………………………………………………………………9

Supplemental Figure 6..…………………………………………………………………10

Supplemental Table 1. Associations of RAP–PAPi Categories With the Primary Endpoint Across Assessment Time Points

	Pre-PVAD		Early Post-PVAD		24h Post-PVAD	
**Status**	**Unadjusted HR**	***P* Value**	**Unadjusted HR**	***P* Value**	**Unadjusted HR**	***P* Value**
RAP < 15 mmHg and PAPi ≥ 0.9	Reference		Reference		Reference	
RAP ≥ 15 mmHg and PAPi ≥ 0.9	0.8 (0.2–2.7)	0.667	1.2 (0.5–3.0)	0.689	2.7 (1.2–6.1)	0.022
RAP < 15 mmHg and PAPi < 0.9	2.9 (1.2–7.1)	0.020	2.2 (1.6–3.1)	< 0.001	1.8 (1.3–2.6)	0.002
RAP ≥ 15 mmHg and PAPi < 0.9	1.3 (0.5–3.4)	0.556	1.7 (1.1–2.7)	0.022	3.1 (2.0–4.8)	< 0.001

Values are presented as HRs (95% confidence interval) for association with mortality or MCS reintroduction.

HR, hazard ratio; MCS, mechanical circulatory support; PAPi, pulmonary artery pulsatility index; PVAD, percutaneous ventricular assist device; RAP, right atrial pressure.

Supplemental Table 2. Associations Between RV Metrics and Mortality Across Assessment Time Points

		Pre-PVAD		Early Post-PVAD		24h Post-PVAD	
**Event**	**RV Metrics**	**Unadjusted HR**	***P* Value**	**Unadjusted HR**	***P* Value**	**Unadjusted HR**	***P* Value**
Mortality	With hRVD	2.1 (0.9–4.9)	0.080	2.1 (1.5–2.9)	< 0.001	2.3 (1.6–3.2)	< 0.001
	RAP ≥ 15 mmHg	0.9 (0.4–2.1)	0.827	1.1 (0.7–1.7)	0.578	2.6 (1.7–3.8)	< 0.001
	PAPi < 0.9	2.5 (1.1–5.5)	0.022	2.1 (1.5–2.9)	< 0.001	2.3 (1.6–3.2)	< 0.001

Values are presented as HRs (95% confidence interval) for association with mortality.

HR, hazard ratio; hRVD, hemodynamic right ventricular dysfunction; PAPi, pulmonary artery pulsatility index; PVAD, percutaneous ventricular assist device; RAP, right atrial pressure; RV, right ventricular.

Supplemental Table 3. Associations Between hRVD Status and Complications During PVAD Support

Complication	All	Groups by RV Metrics 24h Post-PVAD		*P* Value
		Without hRVD (RAP < 15 mmHg and PAPi ≥ 0.9)	With hRVD (RAP ≥ 15 mmHg and/or PAPi < 0.9)	
Composite complication, events/patients (%)	228/436 (52)	129/267 (48)	99/169 (59)	0.036
Hemolysis, events/patients (%)	132/436 (30)	73/267 (27)	59/169 (35)	0.093
Major bleeding, events/patients (%)	165/436 (38)	84/267 (31)	81/169 (48)	< 0.001
Limb ischemia, events/patients (%)	32/436 (7)	17/267 (6)	15/169 (9)	0.327

Values are presented as number of patients (%).

hRVD, hemodynamic right ventricular dysfunction; PAPi, pulmonary artery pulsatility index; PVAD, percutaneous ventricular assist device; RAP, right atrial pressure; RV, right ventricular.

Supplemental Figure 1. Patient Flow

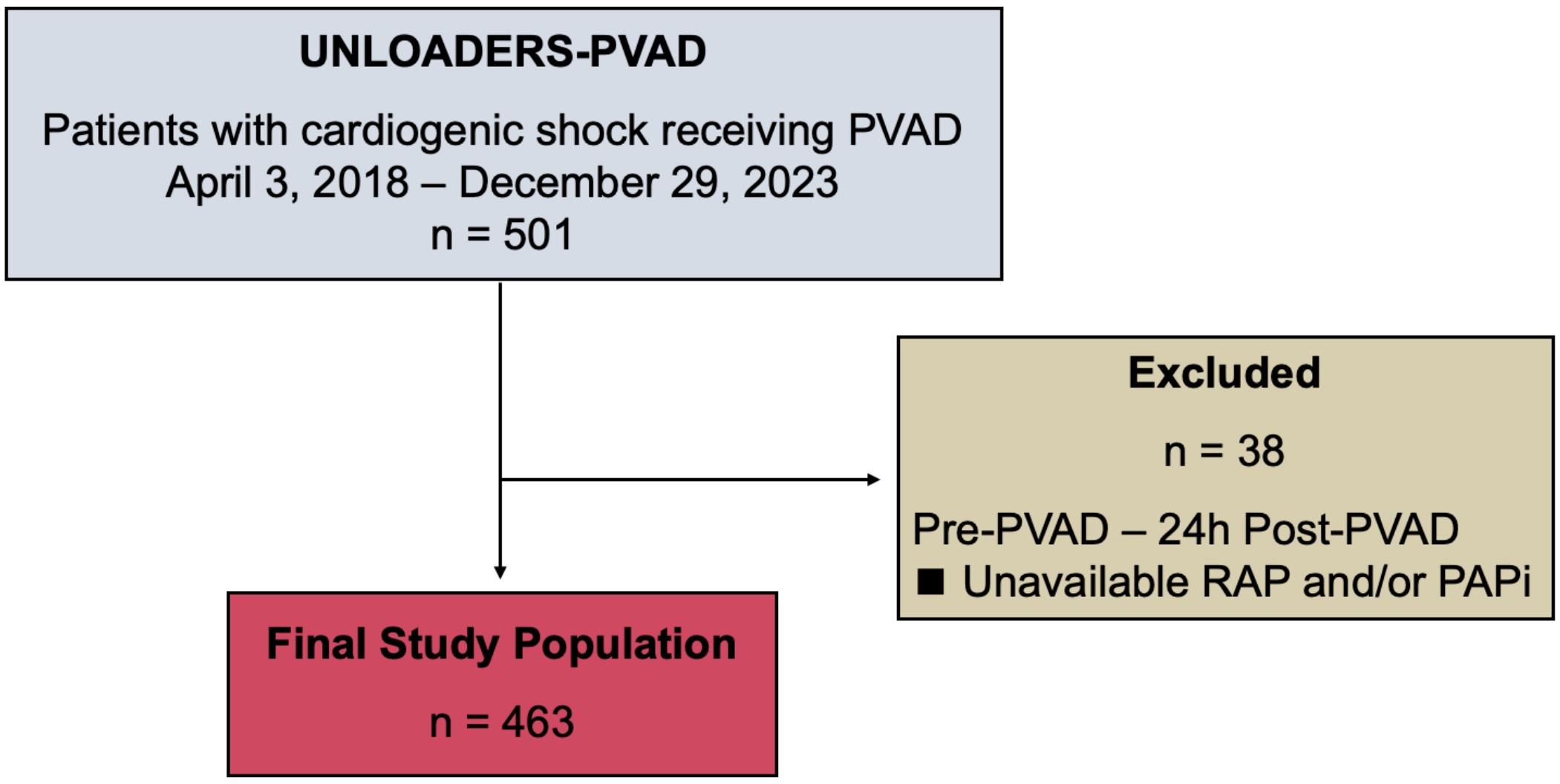

Among the 501 patients enrolled in the UNLOADERS-PVAD registry, those in whom hRVD status could not be assessed at any of the three time points (pre-PVAD, early post-PVAD, and 24h post-PVAD) due to missing RAP or PAPi data were excluded. Consequently, 463 patients were included in the final analysis.

hRVD, hemodynamic right ventricular dysfunction; PAPi, pulmonary artery pulsatility index; PVAD, percutaneous ventricular assist device; RAP, right atrial pressure; UNLOADERS-PVAD, UNLoading and heart recOvery with ADvanced mEchanical ciRculatory Support: optimal management of Percutaneous Ventricular Assist Device.

Supplemental Figure 2. Associations Between RAP and PAPi Across Assessment Time Points, Stratified by Concomitant VA-ECMO

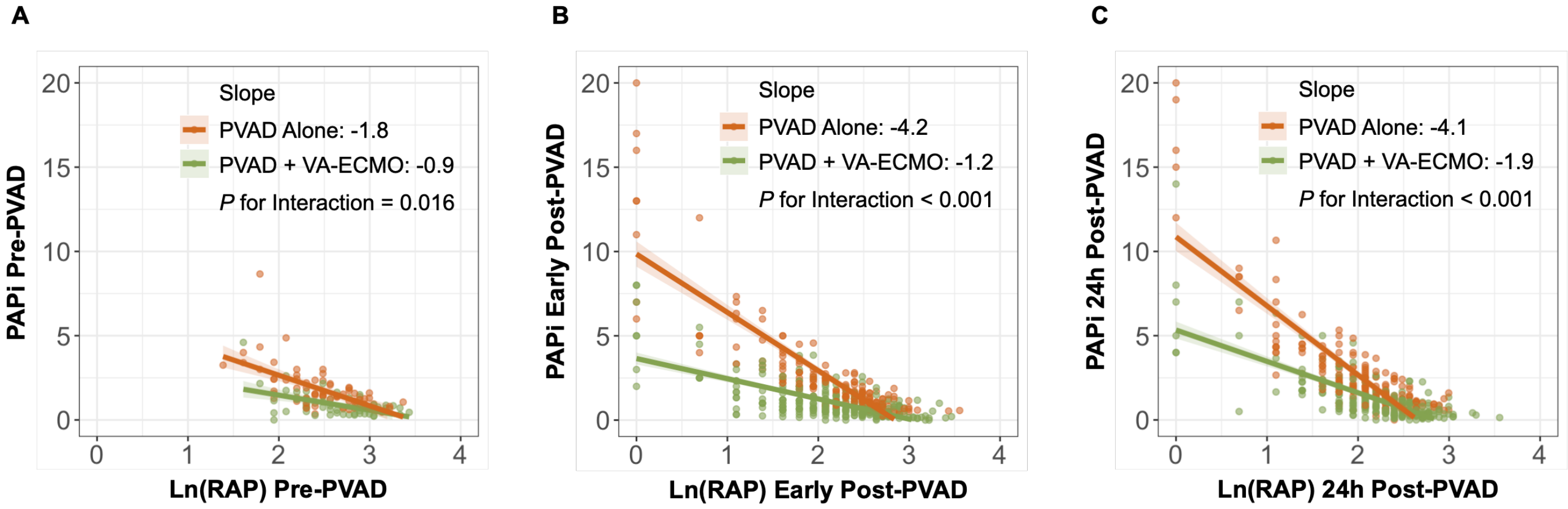

Associations between PAPi and ln(RAP) were assessed pre-PVAD (**A**), early post-PVAD (**B**), and at 24h post-PVAD (**C**). Slopes were estimated, and differences by concomitant VA-ECMO were evaluated using P values for interaction.
Because RAP showed a skewed distribution and a non-linear relationship with PAPi, RAP was log-transformed for the regression analyses.

PAPi, pulmonary artery pulsatility index; PVAD, percutaneous ventricular assist device; RAP, right atrial pressure; VA-ECMO, venoarterial extracorporeal membrane oxygenation.

Supplemental Figure 3. Associations of RAP and PAPi With Event Rates Across Assessment Time Points, Stratified by Concomitant VA-ECMO

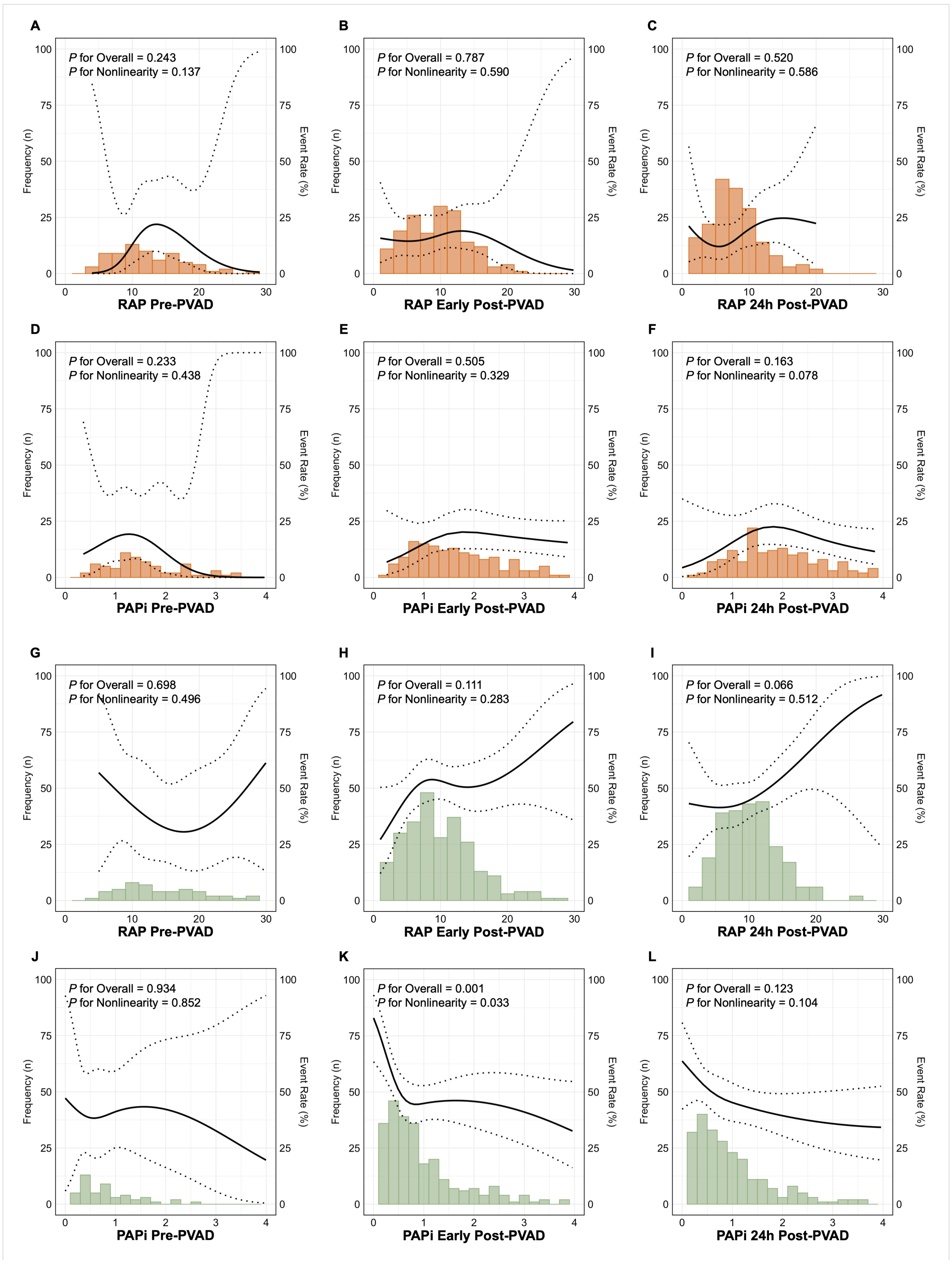

Spline curves illustrating the relationships of RAP (**A**–**C**, **G**–**I**) and PAPi (**D**–**F**, **J**–**L**) with the event rates of the primary endpoint at each assessment time point in patients with PVAD alone (**A**–**F**) and those with PVAD and concomitant VA-ECMO (**G**–**L**).

PAPi, pulmonary artery pulsatility index; PVAD, percutaneous ventricular assist device; RAP, right atrial pressure; VA-ECMO, venoarterial extracorporeal membrane oxygenation.

Supplemental Figure 4. Time-Dependent ROC Curves of RAP and PAPi Across Assessment Time Points for the Primary Endpoint

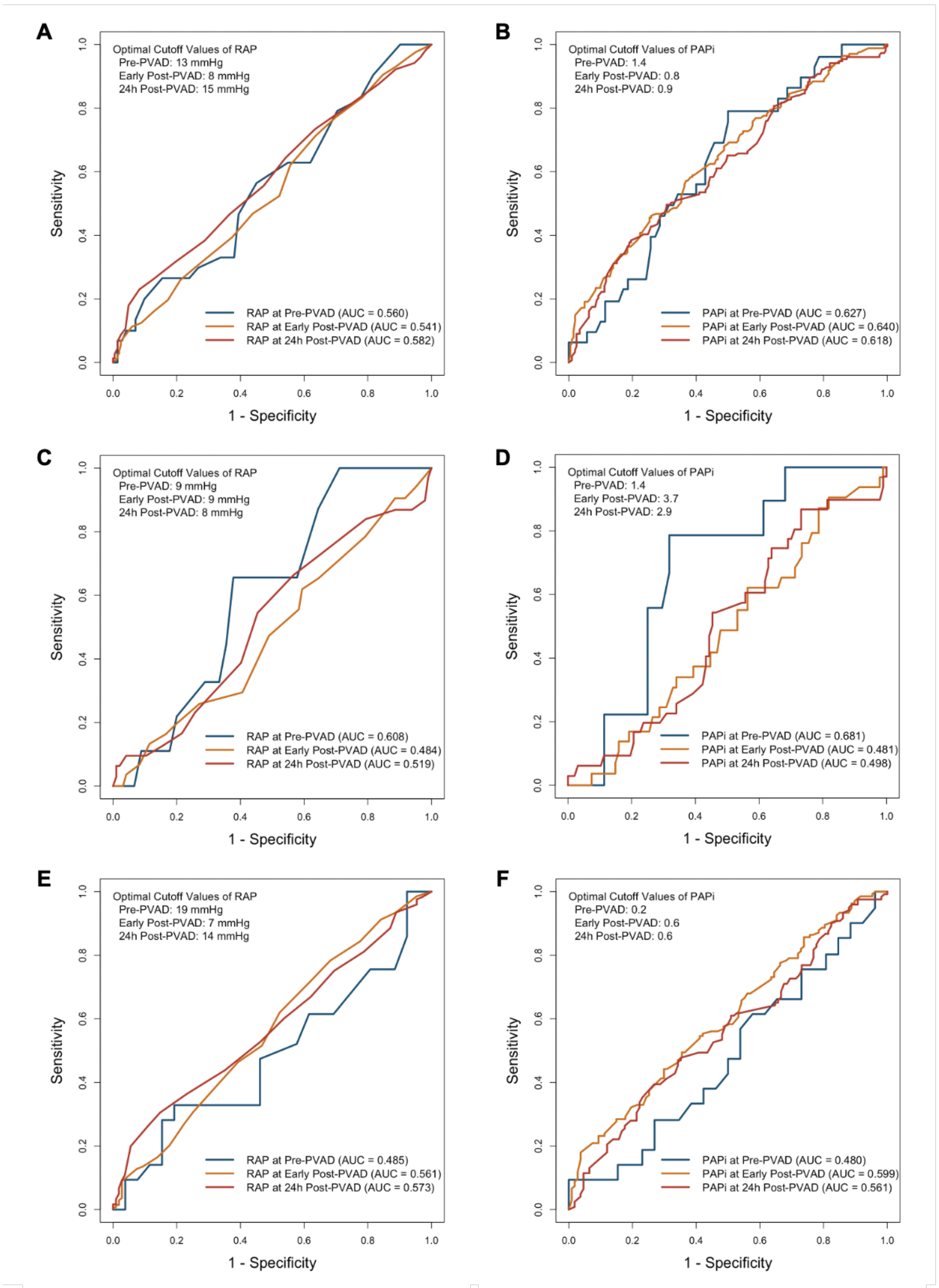

Time-dependent ROC curves of RAP (**A**, **C**, **E**) and PAPi (**B**, **D**, **F**) at each assessment time point for discrimination of the primary endpoint at 30 days. The top panels represent the overall cohort (**A**, **B**), the middle panels represent patients with PVAD alone (**C**, **D**), and the bottom panels represent patients with PVAD and concomitant VA-ECMO (**E**, **F**). Time-dependent AUC values are shown in each panel. Optimal cutoff values were determined using the Youden index.

AUC, area under the curve; PAPi, pulmonary artery pulsatility index; PVAD, percutaneous ventricular assist device; RAP, right atrial pressure; ROC, receiver operating characteristic; VA-ECMO, venoarterial extracorporeal membrane oxygenation.

Supplemental Figure 5. Kaplan–Meier Curves for the Primary Endpoint After Excluding Patients With Prior VA-ECMO Use Before PVAD Implantation

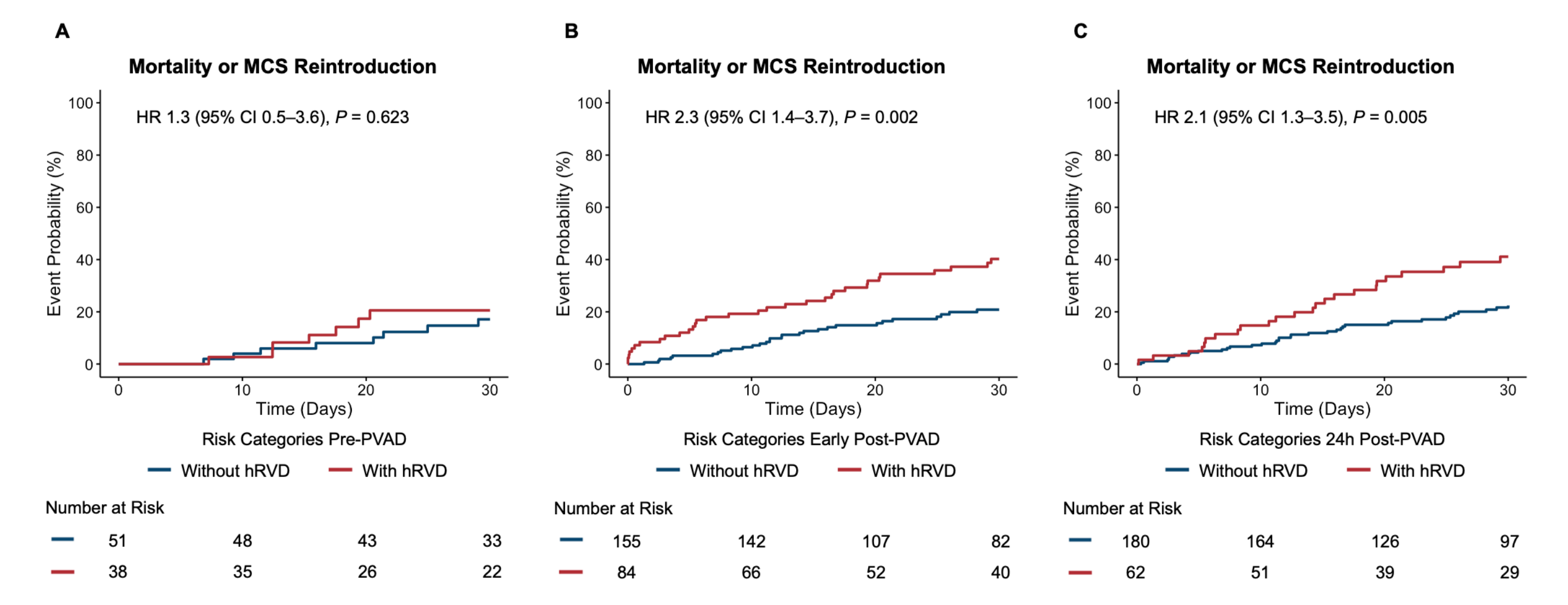

As a sensitivity analysis for the primary endpoint, patients with prior VA-ECMO use before PVAD implantation were excluded from the overall cohort. Accordingly, the analysis was performed in a cohort consisting of patients treated with PVAD alone and those who underwent subsequent VA-ECMO after PVAD implantation.
Kaplan–Meier curves for the cumulative incidence of the primary endpoint stratified by hRVD status at each assessment time point (**A**–**C**).
MCS reintroduction was defined as unplanned initiation of VA-ECMO, PVAD, or intraaortic balloon pump after PVAD discontinuation.

CI, confidence interval; HR, hazard ratio; hRVD, hemodynamic right ventricular dysfunction; MCS, mechanical circulatory support; PAPi, pulmonary artery pulsatility index; PVAD, percutaneous ventricular assist device; RAP, right atrial pressure; VA-ECMO, venoarterial extracorporeal membrane oxygenation.

Supplemental Figure 6. Subgroup Analyses of the Association Between hRVD and Risk of the Primary Endpoint Across Assessment Time Points

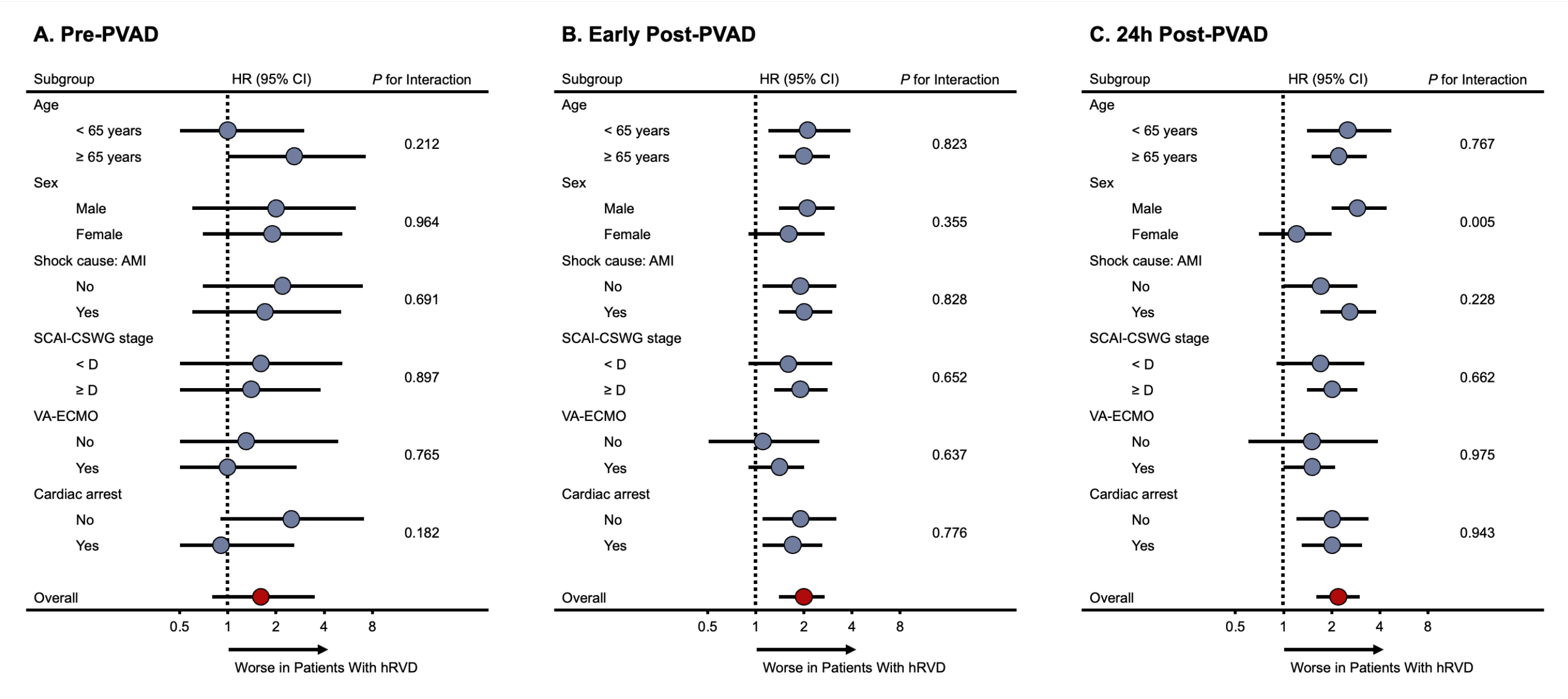

Forest plots showing subgroup analyses of the association between hRVD and the risk of the primary endpoint according to baseline characteristics pre-PVAD (**A**), early post-PVAD (**B**), and at 24h post-PVAD (**C**).

AMI, acute myocardial infarction; CI, confidence interval; CSWG, cardiogenic shock working group; HR, hazard ratio; hRVD, hemodynamic right ventricular dysfunction; PVAD, percutaneous ventricular assist device; SCAI, society for cardiovascular angiography and interventions; VA-ECMO, venoarterial extracorporeal membrane oxygenation.
